# Clinical outcomes following direct anterior approach during total hip arthroplasty without hip extension: a retrospective comparative study

**DOI:** 10.1186/s12891-024-07416-y

**Published:** 2024-04-10

**Authors:** Hua-zhang Xiong, Kuan Xiang, Xiu-qi Liu, Ying Jin, He-he Zhong, Shu-hong Wu, Jia-chen Peng

**Affiliations:** Department of Orthopedic Surgery, Affiliated Hospital of Zunyi Medical University, 149# Dalian Road, Zunyi, 563003 People’s Republic of China

**Keywords:** Direct anterior approach, Total hip arthroplasty, Comparison, Traditional approach, Femoral-release-first

## Abstract

**Background:**

Traditional total hip arthroplasty (THA) using the direct anterior approach (DAA) requires a hip extension. This study aimed to compare the clinical outcomes of patients undergoing THA with DAA using either the no hip extension (NHE) or the traditional hip extension (THE) strategy.

**Methods:**

A retrospective analysis of demographics, clinical and radiological outcomes, and occurrence of complications was performed using data from 123 patients treated between January 2020 and November 2021. The patients were categorised into two groups: NHE (84 patients) and THE (39 patients).

**Results:**

The NHE group exhibited shorter operative time and had more male participants with higher ages. Comparable outcomes were observed in the visual analogue scale, Harris Hip, and Oxford Hip scores at the final follow-up. Furthermore, complications were observed in the NHE and THE groups, including two and one greater trochanteric fractures and three and one transfusions, respectively.

**Conclusions:**

Compared to the THE, employing the NHE strategy during THA with DAA in elderly and young female patients resulted in comparable clinical outcomes with several advantages, such as favourable surgical time. The NHE method also exhibited good safety and effectiveness. Therefore, the NHE strategy may be a favourable option for elderly and young female patients.

## Background

The direct anterior approach (DAA) is a primary surgical approach in total hip arthroplasty (THA) for end-stage osteoarthropathy and has shown satisfactory clinical outcomes [1]. However, achieving appropriate elevation and exposure of the proximal femur, necessary for femoral broach and stem installation, remains technically demanding and is associated with a higher risk of complications, including greater trochanter fractures and muscular impairment [2, 3].

Hip extension using an operation table has emerged as the principal method for elevation and exposure of the proximal femur and has been extensively studied [4–7]. Moslemi et al. [5] reported that using a standard table for performing THA via the DAA does not provide better control over leg length than using a traction table, provided preoperative planning is conducted. Implant placement on a standard table is comparable, with a similar risk of complications. Knoth et al. [4] reported slightly better outcomes when using a standard table for THA compared to an extension table. Given the added expenses, human resources, and time associated with the operation table-related hip extension method for elevating and exposing the proximal femur, opting for the no hip extension (NHE) method remains a viable choice.

In our clinical practice, employing a surgical technique involving NHE can accomplish the femoral procedure, leading to a shorter operative time and fewer human resources. The primary advantages of using NHE during DAA for THA include saving time on the installation of hip extension equipment, enhancing the ability to manipulate the lower limb, intraoperative monitoring of leg length bilaterally (by palpation of the top patella and medial malleoli), testing hip stability, and facilitating intraoperative fluoroscopy. These advantages enable a more simplified operation; thereby, enhancing surgical ease. However, to the best of our knowledge, no comparative studies have investigated the postoperative outcomes of the NHE method and traditional hip extension (THE) through a standard table in patients undergoing THA using DAA.

Therefore, this retrospective study aimed to evaluate the differences in functional and radiological outcomes and complications between the NHE and THE methods in patients undergoing THA using DAA. We hypothesised that the NHE method would provide comparable postoperative outcomes, making it a viable choice for patients.

## Methods

This study was approved by the Ethics Committee of the Affiliated Hospital of Zunyi Medical University (KLL-2023-595). All methods were performed in accordance with the Chinese Ethical Guidelines for Medical and Biological Research Involving Human Subjects. Data for each patient was extracted from their medical records. All surgeries in this study were performed using a DAA with or without hip extension by a senior surgeon at our institution. The NHE group was defined as patients undergoing DAA THA without hip extension, whereas the THE group comprised those undergoing DAA THA with hip extension. The learning curves for both methods were completed based on a previously defined learning curve [8, 9].

The inclusion criteria were as follows: end-stage hip osteoarthropathy, age 20–80 years, body mass index (BMI) < 30 kg/m^2^ [10], and American Society of Anaesthesiologists (ASA) grade < 4. This study met the diagnostic criteria for hip osteoarthritis (OA) provided by the Guidelines for the Diagnosis and Treatment of OA [11]. The exclusion criteria included: a history of single-stage bilateral surgery, hip surgery or trauma, lumbar spinal fusion, serious acetabular defects, high-grade developmental dysplasia of the hip (> grade II), serious organic or infectious diseases, lack of complete imaging data, loss to follow-up, and the presence of other prostheses.

All surgical notes were carefully reviewed to identify the surgical technique used (NHE or THE). Patients who underwent treatment between January 2020 and November 2021 were assessed for inclusion in this study. All the included patients received the ACT cup-ML-TH stem system (AK, Beijing, China) for THA. Cementless press-fit components were uniformly applied to the acetabulum and femur. Using standardised prostheses allowed us to eliminate any additional procedures required for the initial THA, facilitating the creation of comparable groups for analysis.

## Surgical technique

**Fig. 1:**
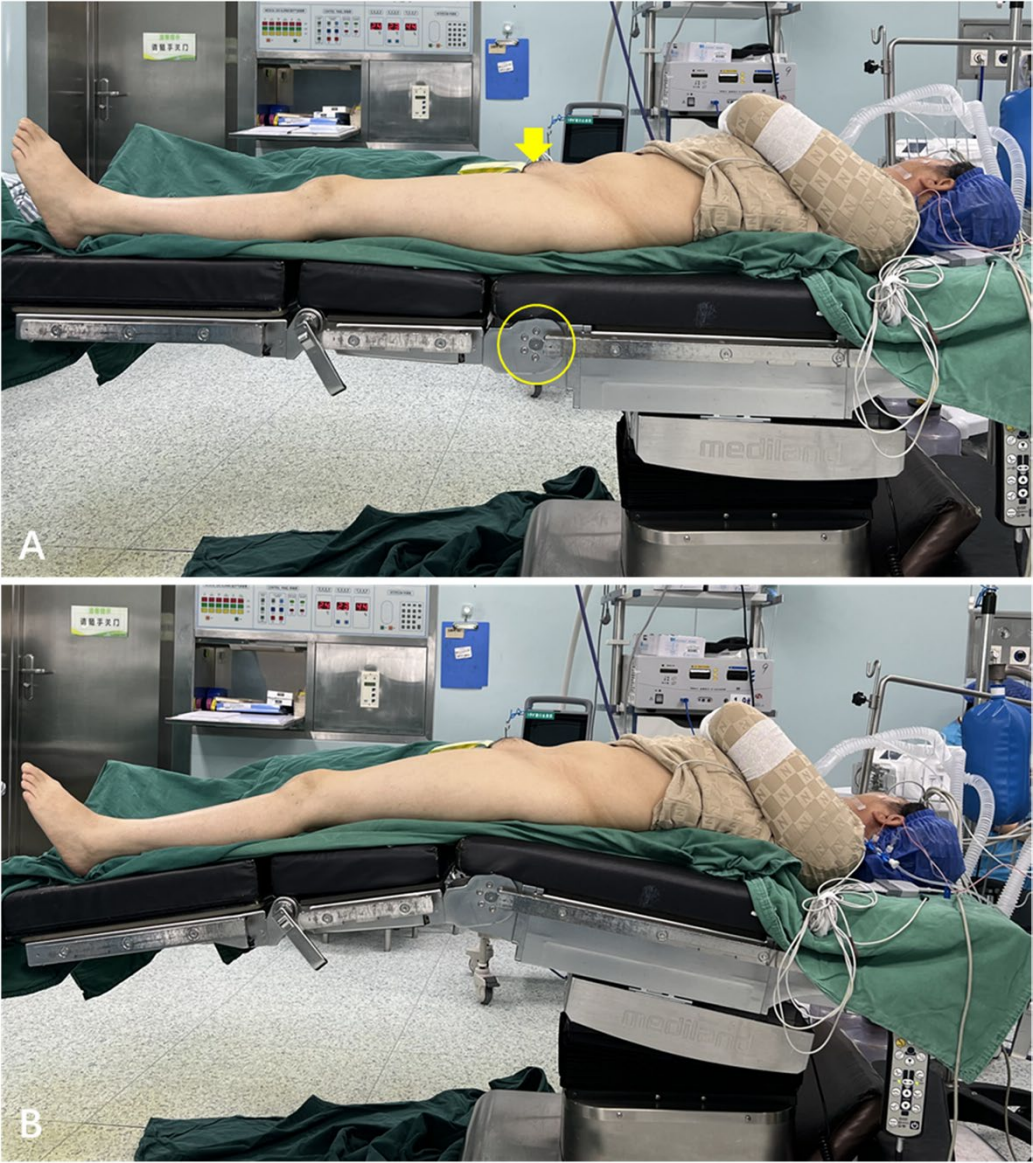
**A** No hip extension and intraoperative lower limb positioning in the NHE group, with the symphysis pubis (yellow arrow) placed at the flexion point (inside the yellow circle) of the operating table. **B** Positioning of the involved limb with hip extension when preparing the proximal femur in the THE group. NHE, no hip extension; THE, traditional hip extension

All patients underwent general anaesthesia and had well-controlled blood pressure. All surgeries were performed with the patient in a supine position on an orthopaedic table under fluoroscopy. The affected hip was placed on the far ipsilateral side of the table, and a levelled pelvis was achieved. The symphysis pubis was placed at the flexion point of the operating table (Fig. 1A). In cases where femoral procedures could not be completed using the NHE method, conversion to the THE method with hip extension was performed (Fig. 1B). The flexion point of the operating table facilitated hip joint extension to improve the angle of insertion of the femoral component and instruments during the femoral procedure in patients who were converted to the THE method.

**Fig. 2:**
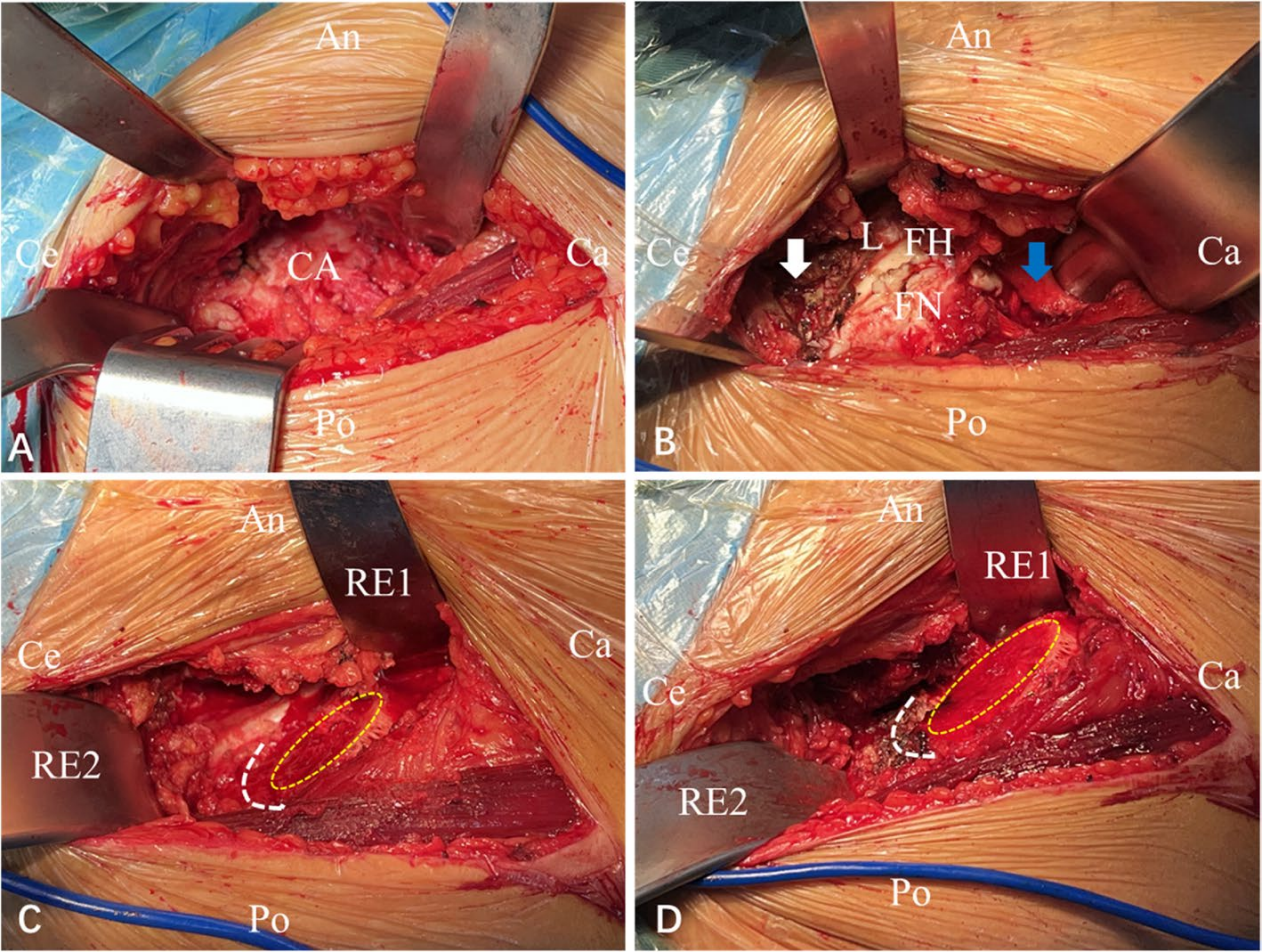
**A** Exposure of the anterior joint capsule. **B** Release of the reflected head of the rectus (white arrow), removal of the anterior joint capsule, and release of the inferomedial pubofemoral ligament initiating from the lesser trochanter (blue arrow). **C** Intraoperative image showing the location of the femoral neck (yellow dotted line) before release. The white dotted line shows the area of release, including the posterior superior capsule, the superior capsule, and the conjoined tendon. **D** Intraoperative image showing the location of the femoral neck (yellow dotted line) after release of the proximal femur (white dotted line)

**Fig. 3:**
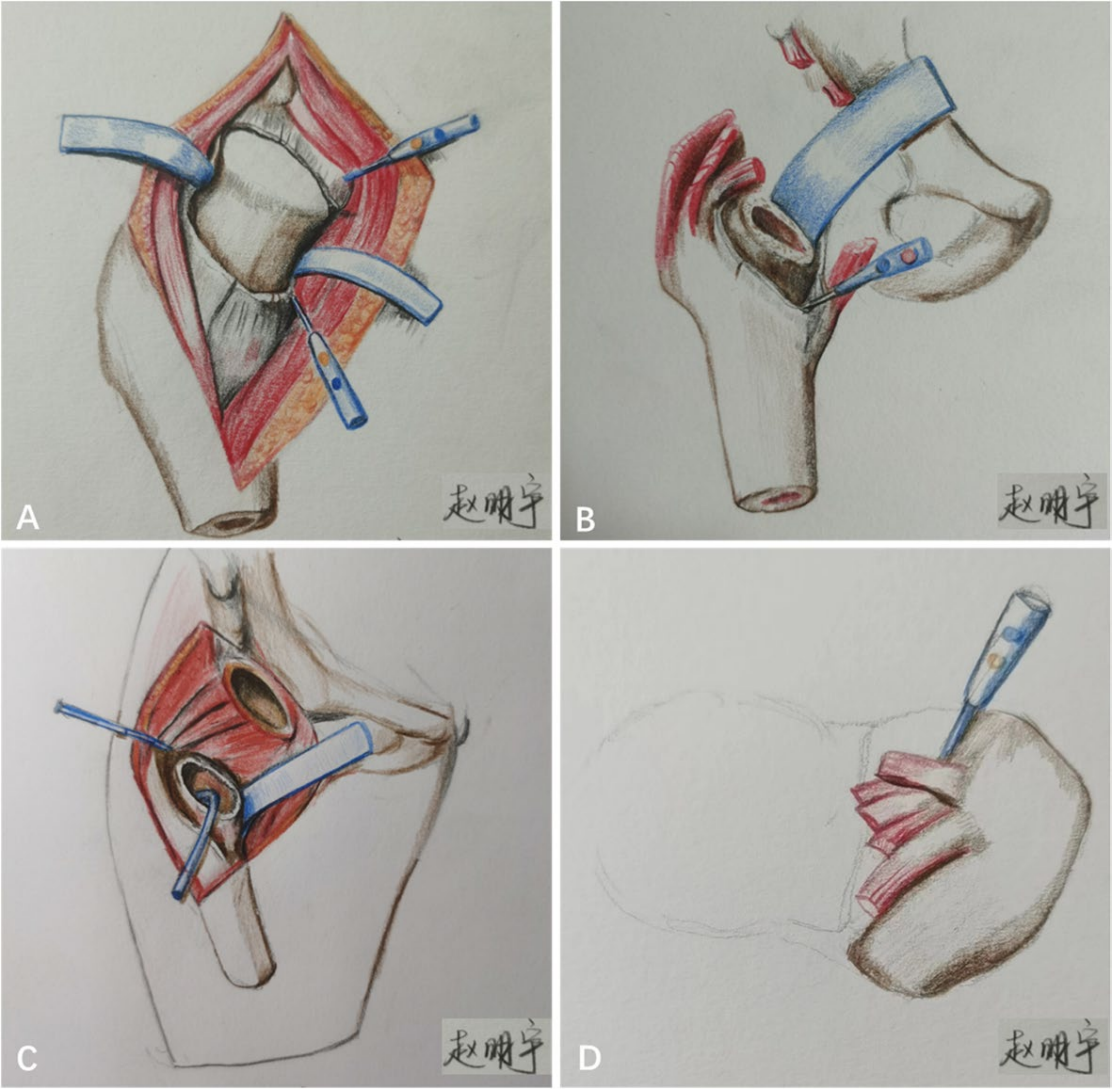
**A** Release of the reflected head of the rectus, excision of the anterior joint capsule. **B** Release of the inferomedial pubofemoral ligament initiating from the lesser trochanter. **C** The schematic drawings show the area of release, including the posterior superior capsule, the superior capsule, and the conjoined tendon. **D** Release of the conjoined tendon from the greater trochanter

All patients were operated through a DAA according to the method described in our previous study [12]. An incision was made approximately 2 cm distal and 2 cm lateral to the anterior superior iliac spine, which extended in the direction of the fibular head and was approximately 8–10 cm in length. The fascia of the tensor fasciae latae (TFL) muscle was split along the muscle fibres to allow blunt separation and access to the Hueter gap between the sartorius and TFL muscles. The ascending branch of the lateral femoral circumflex artery was carefully ligated, the anterior capsule was exposed (Fig. 2A), and the reflected head of the rectus was released to improve exposure (Fig. 2B). A capsulectomy was performed, the capsule dissection was initiated in line with the femoral neck, the inferior medial and superior lateral capsular femoral flaps were removed (Fig. 2B), and the capsule excision was considered the primary release (Fig. 3A). Subsequently, the lesser trochanter initiated release of the pubofemoral ligament inferomedially (Figs. 2B and 3B). The femoral neck was cut, and the femoral head was removed. The affected limb was first positioned for external rotation and adduction. A Mueller retractor (RE1; Fig. 2C and D) was placed over the posterior aspect of the femoral neck to distract the medial tissues. Another Mueller retractor (RE2; Fig. 2C and D) was positioned over the superior site of the greater trochanter with the hip abductor muscles behind it to allow separation of the muscle and joint capsule and to improve the appropriate exposure of the proximal femur. A bone hook was placed in the femoral canal to distract the proximal femur towards the anterolateral aspect of the incision. The residual pubofemoral ligament, posterior capsule, superior capsule, and conjoined tendon were gradually released (Figs. 2C-D and 3C-D). Once the mouth of the femoral neck was above the anterior rim of the acetabulum, which permitted sufficient exposure for broaching and stem installation (Fig. 2D), the femoral release was completed. Acetabular and femoral procedures were performed according to the preoperative planning.

The acetabular cup procedure was performed routinely. A retractor was placed posterior to the acetabulum for retracting the femur posteriorly. A curved retractor was placed on the anterior rim of the acetabulum to retract the iliopsoas, rectus femoris, and sartorius anteriorly. Another retractor was placed anteroinferiorly into the obturator foramen to expose the inferior acetabular margin. After the 360-degree acetabular visualization was achieved, the labrum was resected. Subsequently, acetabular reaming was performed using reamers to widen the acetabular rim until a satisfactory press fit was obtained. The final component was then impacted into the acetabulum with adjunctive screw fixation, followed by the installation of the acetabular liner.

**Fig. 4:**
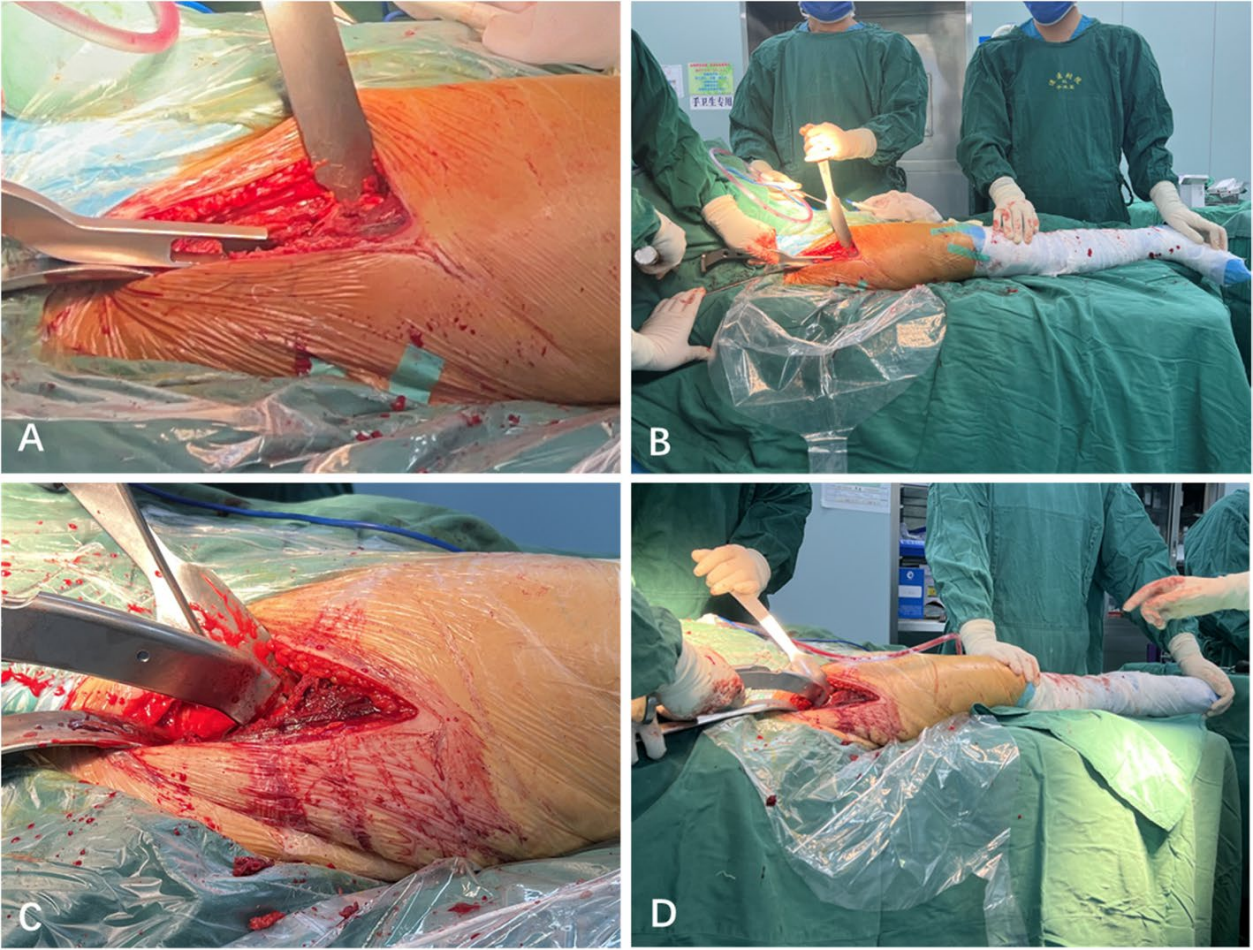
Intraoperative close-up (**A**) and long-shot (**B**) images showing the proximal femur grooved (**A**, **B**) without hip extension in the right total hip arthroplasty. Intraoperative close-up (**C**) and long-shot (**D**) images showing the proximal femur broached

**Fig. 5:**
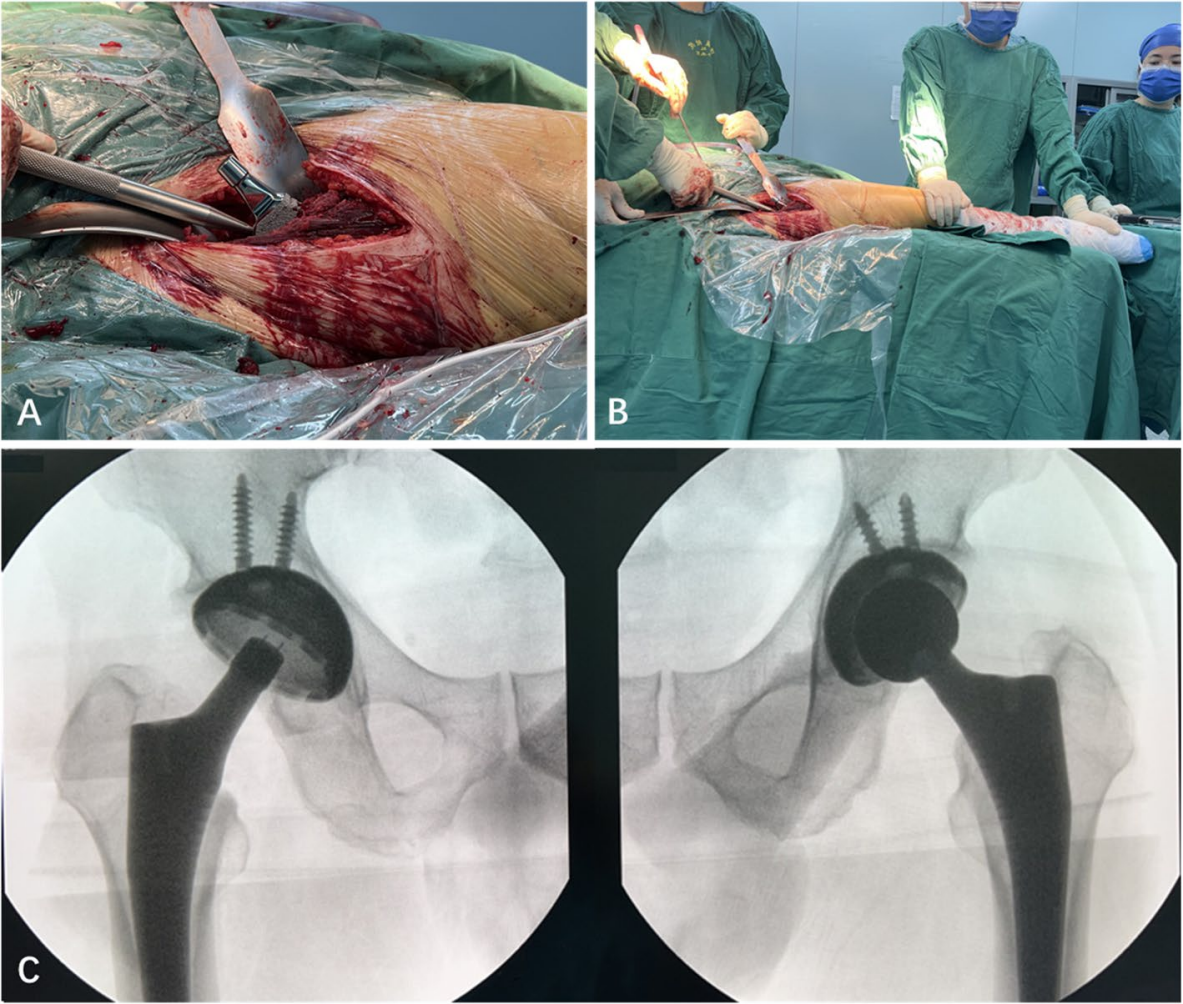
Intraoperative close-up (**A**) and long-shot (**B**) image showing the femoral stem installation without hip extension requirement. Intraoperative imaging showing good acetabular and femoral alignment with the same lower limb length in the right total hip arthroplasty (**C**)

The involved leg was then positioned for external rotation and adduction, allowing the surgeon to determine the appropriate elevation height of the proximal femur. If instrument insertion was unaffected (Fig. 2D), subsequent femoral procedures were performed using the NHE technique. The proximal femur was broached to an appropriate size (Fig. 4A-D), and the femoral implant was installed (Fig. 5A-B) from preoperative templating. Head trial components were installed, and the hip joint was reduced. After hip repositioning, stability was evaluated at the maximum external rotation of extension and maximum internal rotation of flexion. The affected leg length was assessed by palpation of the medial malleoli and top patella. Subsequently, fluoroscopy was performed to assess the involved leg length, component positioning, and the presence of iatrogenic fractures around the components (Fig. 5C). Subsequently, the definitive femoral implants were installed.

In contrast, if the instruments of the femoral procedure were affected and the femoral procedure could not be accomplished using the NHE technique after evaluation, the THE method was chosen. Subsequently, the involved leg was positioned for external rotation and adduction with hip extension using an orthopaedic table. Femoral procedures were performed as described for the NHE group. The femoral trial components were installed, and the trial component and orthopaedic table were repositioned. Hip stability was evaluated at maximum external rotation of extension and maximum internal rotation of flexion. The affected leg length was assessed by palpation of the medial malleoli and top patella. Subsequently, fluoroscopy was performed to assess the involved leg length, component positioning, and the presence of iatrogenic fractures around the components. The involved leg was then positioned for external rotation and adduction with hip extension, and definitive femoral implants were installed.

The sizes and positions of the femoral stem and the acetabular cup were confirmed radiographically. The acetabular cup was placed in a safe zone of 30–50° inclination and 5–25° anteversion [13]. The incision was sutured layer by layer, and no drainage tubes were used.

## Perioperative management

All patients received pain management, prophylactic antibiotics, and postoperative prophylactic antithrombotics. Hip mobility was permitted, and ambulation was initiated 1 day postoperatively. Discharge to home was aimed for on postoperative day 2. Haemoglobin (Hb) levels were measured within 24 h postoperatively, and a blood transfusion was required if the Hb level was < 70 g/L. Follow-ups were performed according to our institution’s standard postoperative schedule at 1, 2, 3, 6, and 12 months, and annually afterwards.

## Data collection

### Demographic and clinical characteristics

Patients’ data were collected, including sex, age, BMI, ASA grade, involved side, Hb drop, blood transfusion, operative time, and postoperative hospital stay. The operative time was measured from the initiation of skin incision to the completion of incision suturing. The Hb drop was calculated as the preoperative Hb value minus the value on postoperative day 1.

### Clinical outcomes

Clinical outcomes were evaluated using the Harris Hip Score (HHS) [14], Oxford Hip Score (OHS) [15], and visual analogue scale (VAS) score [16]. The HHS was used to assess hip function recovery, with scores ranging from 0 (worst) to 100 points (best). The OHS was used to evaluate hip pain and function, with scores ranging from 0 (worst) to 48 (best). The VAS score was used to assess pain on a scale of 0–10 (0 = no pain and 10 = worst pain). Postoperative patient-reported outcomes were recorded and analysed to compare differences between the two surgical strategies.

### Radiographic evaluations

All patients underwent routine anteroposterior hip radiographs preoperatively, 1 day postoperatively, and 3 months postoperatively, using a standardised technique [17]. A position with a 20° internal rotation of the hip joint was used to achieve a standardised and reproducible image during follow-up. The X-ray tube was placed perpendicularly at a 1-m distance from the table. Radiographs obtained 3 months postoperatively were used to evaluate stem alignment (graded as varus, neutral, or valgus) and cup alignment (inclination and anteversion angles) [18, 19]. The inter-teardrop line was used as the reference line for measuring the acetabular cup inclination angle, and a deviation > 3° from the axis of the femur was defined as valgus or varus position [20]. Leg length discrepancy (LLD) was assessed, and the goal of the length of the patient’s involved limb was equal to the length of the contralateral limb. An equal length was defined as an LLD between − 10 mm and 10 mm [5]. The periprosthetic radiolucent lines and osteolysis were assessed in the femur according to the 14 zones of Gruen [21] and in the acetabulum according to DeLee and Charnley [22, 23]. Subsidence of the femoral stem was defined as any change in distance between the stem shoulder and the tip of the greater trochanter on the final follow-up radiographs compared with immediate postoperative radiographs [24]. Femoral stem loosening was defined as subsidence > 5 mm [25], progressive femoral stem tilt [26], radiolucent lines > 2 mm at the bone-stem interface [21], or multiple bone cavitations [26, 27]. Acetabular cup loosening was defined as a tilt > 5° or radiolucent lines > 2 mm at the bone-component interface in two or three DeLee and Charnley zones on the final follow-up radiographs compared to the immediate postoperative radiographs [21, 22]. All radiographs were evaluated by two independent radiologists blinded to the clinical treatment and outcomes. Inter-observer reliability between the two radiologists was evaluated using interclass correlation coefficients (ICCs) interpreted as follows: > 0.9: excellent; 0.75–0.9: good; 0.50–0.74: fair; and < 0.50: poor. ICCs were interpreted using previously reported semi-quantitative criteria [28].

### Perioperative complications

Data on complications, including greater trochanter fractures, anaemia, lateral femoral cutaneous nerve injury, incision-related conditions (oozing, delayed healing, and infection), dislocation, and venous thromboembolism (VTE), were collected.

### Statistical analyses

The sample size for this study was calculated using Slovin’s formula, as previously described by Ellen [29]. The participants’ size was determined according to the number of patients with hip OA reported by Li et al. [30]; N was 60 patients for 6 months, and e was 0.05 at a 95% confidence interval; 60 patients were required for the present study. Each group consisted of at least 30 patients. Statistical tests were performed using SPSS® software version 22 (SPSS Inc., Chicago, IL, USA) by a researcher blinded to surgical procedures and data collection. All values were expressed as means with standard deviations. The Kolmogorov–Smirnov test was performed for each continuous variable to determine normality. The Mann–Whitney U test was used for continuous variables. Differences between sets of categorical data were analysed using Fisher’s exact probability or Pearson’s chi-square test for outcomes between the NHE and THE groups. Statistical significance was set at *p* < 0.05.

## Results

### Patients

**Fig. 6:**
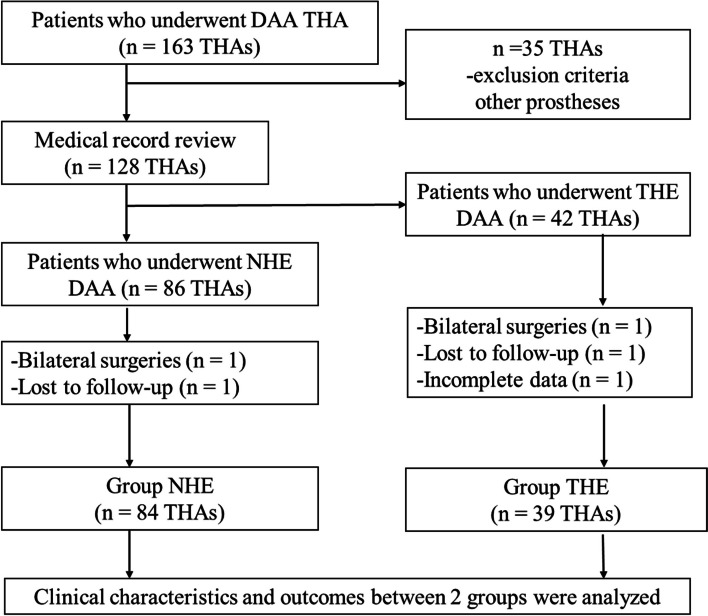
Flowchart of patient enrolment. DAA, Direct anterior approach; NHE, No hip extension; THE, Traditional hip extension; THA, Total hip arthroplasty

Of the initial 163 screened patients, 35 were excluded based on the exclusion criteria (Fig. 6). Subsequently, we conducted a retrospective review of 123 patients who underwent THA with DAA between January 2020 and November 2021. Each patient required a minimum follow-up of 24 months for inclusion in this study.

Of the 123 THAs, 84 (68.3%) were performed using the NHE method, whereas 39 (31.7%) were performed using the THE. Demographic data, including BMI, ASA grade, involved side, postoperative hospital stay, Hb level drop, and total follow-up time, showed no significant differences between the two groups. Additionally, age, sex, and operative time showed significant differences between both groups (*p* < 0.05) (Table 1).

**Table 1:** Demographics and characteristics of patients^ψ^

Characteristic	NHE group	THE group	*P* Value
Number	84 (68.3%)	39 (31.7%)	
**Age (y)**	**58.7 ± 12.0**	**50.3 ± 8.8**	**0.000**^**a**^
**Sex (M/F)**	**32/52**	**31/8**	**0.000**^**b**^
M	38.1%	79.5%	
F	61.9%	20.5%	
Body mass index (kg/m^2^)	24.4 ± 2.1	25.2 ± 2.3	0.081^a^
ASA grade (I/II/III)	57/21/6	26/10/3	0.310^b^
I	67.9%	66.7%	
II	25.0%	25.6%	
III	7.1%	7.7%	
Affected side (right/left)	45/39	20/19	0.848^b^
**Operative time (Mins)**	**56.1 ± 6.2**	**60.1 ± 5.0**	**0.000**^**a**^
Postoperative hospital stay (Days)	2.9 ± 1.0	3.1 ± 1.0	0.410^a^
Follow-up time (Months)	33.3 ± 5.4	34.6 ± 6.0	0.280^a^
Hb drop (g/L)	31.3 ± 6.3	32.3 ± 7.9	0.787^a^

^ψ^Continuous variables are expressed as the mean and standard deviation. Categorical variables are presented as numbers with percentages in parentheses

^a^Independent‑sample Mann–Whitney U test

^b^Fisher’s chi-square test. The boldface indicates statistical significance

### Clinical outcomes

The postoperative outcomes of the HHS, OHS, and VAS scores were comparable between the NHE and THE groups. These differences were not statistically significant (*p* > 0.05) (Table 2).

**Table 2:** Postoperative functional outcomes in patients undergoing NHE or THE DAA for THA^ψ^ at last follow-up

Outcome	NHE group (*N* = 84)	THE group (*N* = 39)	*P* Value
VAS	0.77 ± 0.65	0.69 ± 0.61	0.535^a^
HHS	93.4 ± 3.2	94.4 ± 3.4	0.054^a^
OHS	42.7 + 1.8	43.0 ± 2.6	0.124^a^

^ψ^Continuous variables are expressed as the mean and standard deviation

^a^Independent‑sample Mann–Whitney U test

### Radiological outcomes

No significant differences were observed in LLD between the NHE and THE groups based on the 3-month radiographs. No significant differences in cup inclination or anteversion alignment were observed between the two groups, and no signs of cup migration were observed in either group. Similarly, no significant differences were observed in the varus, neutral, or valgus alignment of the femoral stem between the NHE and THE groups (Table 3). No radiological evidence of femoral stem loosening was observed in either group. The ICCs for the anteversion and inclination of the acetabular cup, alignment (varus, neutral, and valgus), subsidence of the femoral stem, and LLD were 0.986, 0.972, 0.900, 0.979, and 0.977, respectively.

**Table 3:** Postoperative radiological measurements and complications between two groups^ψ^

Measurements	NHE group (*N* = 84)	THE group (*N* = 39)	*P* Value
Acetabular cup			
Inclination angle (°)	41.5 ± 2.2	40.8 ± 2.9	0.308^a^
Anteversion angle (°)	16.0 ± 3.2	17.1 ± 4.1	0.328^a^
Femoral stem			
Varus/ Neutral/ Valgus	1/79/4	1/37/1	0.841^b^
Varus	1.2%	2.6%	
Neutral	94.0%	94.9%	
Valgus	4.8%	2.5%	
Subsidence (mm)	0.95 ± 0.62	0.82 ± 0.60	0.308^a^
Leg length discrepancy	-0.30 ± 3.0	-0.33 ± 2.7	0.716^a^
Complications			
Greater trochanter fracture	2 (2.4%)	1 (2.6%)	1.000^b^
Blood transfusion	3 (3.6%)	1 (2.6%)	1.000^b^

^ψ^Continuous variables are expressed as the mean and standard deviation. Categorical variables are presented as numbers with percentages in parentheses

^a^Independent‑sample Mann–Whitney U test

^b^Pearson’s chi-square test

### Perioperative complications

Complications were observed in the NHE and THE groups, including two (2.4%) and one (2.6%) greater trochanteric fractures and three and one transfusions (Hb < 70 g/L), respectively (Table 3). The overall greater trochanteric fracture rate was 2.4% (3/123). Three patients received blood transfusions due to the postoperative hypohemoglobin. No significant differences in the complications were noted between the NHE and THE groups. No other postoperative complications, such as delayed incision healing, dislocation, VTE, or infection, were observed.

## Discussion

This study showed that NHE DAA THA has notable advantages over THE regarding operative time, with comparable outcomes in clinical and radiographic measurements between the two groups.

Patients in the NHE group were less likely to be male and older; however, no association was observed with BMI. These patients did not require a hip extension, potentially attributed to muscle relaxation. Evaluations were performed thrice before determining the necessity of employing hip extension. Firstly, the strength and volume of the patient’s muscles were assessed; strong patients, such as athletes, manual workers, professional soldiers, and young men, may not be candidates for the NHE method, which could be confirmed by the THE group with more male and young patients. Secondly, after the femoral release was complete, we assessed the height at which the proximal femur could be raised; if the proximal femur was not deep from the body surface, the NHE method might be appropriate. Thirdly, when the instruments of the femoral procedure were blocked or affected by femoral elevation, we converted the NHE to THE. Furthermore, no statistically significant difference was observed in the BMI between the two groups, possibly because the overall BMI in this study was less than 30.

DAA for THA using intermuscular planes has been associated with less perioperative pain, less muscle damage, and rapid recovery [31, 32]. Despite the advantages of DAA, comparative studies evaluating the postoperative outcomes of the NHE and THE methods for DAA are lacking. This study addressed this knowledge gap, and we found similar primary clinical results between the NHE and THE methods for DAA postoperatively, including pain and functional scores (VAS, HHS, and OHS). Similarly, this study showed that similar acetabular cup and stem alignment and LLD were achieved without significant cup migration or stem subsidence between the two groups. This suggests comparable functional recovery between the two methods postoperatively.

The major advantages of the NHE method are the lack of a hip extension requirement and the shorter surgical time. Furthermore, it does not require special surgical instruments or complicate the procedure. NHE has a shorter operative time and may not require hip extension using an orthopaedic table, an important factor affecting clinical outcomes. Notably, several studies have reported that a longer operative time is associated with increased blood loss [29], whereas a shorter operative time is associated with reduced length of hospital stay and risk of readmission [30, 33]. In this study, although the NHE group had a shorter operative time, the postoperative hospital stay, haemoglobin drop, and transfusion rate were not significantly different, which may be related to the shorter overall operative time. In a high-volume THA department (approximately 600 THAs annually), this equates to approximately 2400 min (40 h) of additional surgical time per year. Moreover, our centre estimates an additional 10 min for the installation and patient positioning of an orthopaedic table for hip extension. Extra surgical staff and the higher costs associated with specialised orthopaedic tables increase healthcare costs. The NHE strategy may be a favourable option for improving surgical ease and shortening operative time.

Studies have reported that intraoperative greater trochanteric fractures were observed, possibly due to forced elevation of the proximal femur with incomplete release during surgery [3, 4]. Rueckl et al. [3] reported that routine release of the conjoint tendon during the DAA for THA was associated with a lower risk of greater trochanter fracture than “release-on-demand” for the external rotator tendon. Knoth et al. [4] speculated that this higher rate of greater trochanteric fractures resulted from the larger lever arm required to expose and elevate the proximal femur to insert the femoral stem and broaching instruments. In this study, the overall greater trochanter fracture rate was 2.4%, within the range reported in previous studies, which was between 1.5% and 3.1% [4, 34, 35]. We found two principal causes of greater trochanteric fractures. Firstly, patients with osteoporosis experienced greater trochanteric avulsion fractures as the bone hook pulled the proximal femur anterosuperiorly during femoral release. Secondly, when the proximal femur was broached, the shoulder of the reamer impinged the greater trochanter, resulting in a fracture as the large reamer was withdrawn. Therefore, in patients with osteoporosis, violent lifting of the proximal femur using bone hooks without proper release can be avoided to cause greater trochanteric avulsion fractures. Attention should be paid to the grooving of the proximal femur. If the groove is too shallow, the shoulder of the large reamer may impinge the greater trochanter and cause fracture during withdrawal. Shallow grooving may be related to the absence of hip extension or a deep proximal femur. Valgus alignment of the stem in the NHE group demonstrated a high incidence rate and did not achieve significant differences, which may be related to the shallow grooving of the proximal femur. Hence, the position with or without a hip extension should be carefully evaluated to avoid complications in the femoral procedure, and femoral grooving should be adequate. If femoral elevation is insufficient after release, the hip extension position (THE technique) should be chosen.

In this study, the incidence rates of intraoperative greater trochanteric fractures and transfusions were similar between the two groups. No hip dislocations were observed in either group. In our experience, after excision of the anterior capsule and release of the pubofemoral ligament, posterior capsule, superior capsule, and conjoined ligament, THA can be performed using the DAA with hip adduction, external rotation, and hip extension (THE method) without the need for additional releases. In both groups, the femurs of all patients were released using the above methods to avoid instability caused by excessive release. After completing the acetabular cup procedure, the involved leg was positioned for external rotation and adduction, and the surgeon determined the height at which the proximal femur could be elevated. If instrument insertion was not affected, subsequent femoral procedures were performed using the NHE technique. In contrast, if the instruments used in the femoral procedure were affected after the evaluation, the THE method was chosen. The range of release was consistent in both groups, potentially explaining the absence of differences in postoperative joint instability. Furthermore, we refrained from forcibly pressing down the retractor to elevate the proximal femur to perform the NHE method to cause a TFL injury. These comparable outcomes in functional scores, radiographs, and complications confirmed that NHE was safe and effective.

The exact difference between the two methods was with and without extending hip joints using an orthopaedic table in this study. This study had several unique attributes. Firstly, we evaluated the postoperative outcomes of the novel NHE method for DAA. Secondly, the NHE method for DAA was related to sex and age. Thirdly, comparable functional outcomes were observed between the NHE and THE methods postoperatively. Fourthly, the NHE method for DAA resulted in a shorter surgical time. Finally, the NHE method did not increase the technical requirements and complications, demonstrating good safety and effectiveness.

This study had several limitations. Firstly, this was a retrospective, single-centre study with a small sample size, which is an intrinsic limitation that might introduce bias. However, this retrospective study offered a valuable method for analysing and summarising existing clinical data. The outcomes of the present study may have an important effect on clinical practice and are encouraging. Secondly, the outcomes can be affected by many factors when the techniques are used by other teams or institutions. For example, the patient population may vary by race; patients in South China may have a slender figure, which could result in better exposure of the proximal femur. This may not be appropriate for patients of a strong ethnicity or race. Therefore, like other surgical methods, the NHE method also has a scope of application. Indications for primary THA include a painful hip from OA, osteonecrosis of the femoral head, posttraumatic arthritis, rheumatoid arthritis, congenital/adult hip dysplasia (≤ grade II) with secondary arthritis, acute traumatic fracture of the femoral head or neck. Contraindications include a large posterior acetabular defect, significant preoperative heterotopic ossification (except anterior heterotopic ossification), BMI ≥ 30 kg/m^2^ [10], high grade of developmental dysplasia of the hip (> grade II), serious organic or infectious diseases. Thirdly, during the development of the NHE method, the study groups were small; therefore, the relevant conclusions drawn regarding the complication rates (fracture, anaemia, or infection) may be inaccurate. Further confirmation of the safety and effectiveness of this method requires a large-sample, prospective, randomised, controlled, multicentre clinical study comparing the NHE method and THE methods to ascertain which surgical procedure offers the best efficacy. Fourthly, the procedure was performed over a short period. The experience of the surgeon and the team may have partially affected the outcomes. Finally, this study only evaluated the results of a single prosthesis. The selection range of the prosthesis was small, and the results were not compared with other prostheses. We intend to increase the number of prostheses used in future studies.

## Conclusions

Compared to the THE, employing the NHE strategy during THA with DAA in elderly and young female patients resulted in comparable clinical outcomes with several advantages, such as favourable surgical time. The NHE method also exhibited good safety and effectiveness. Therefore, the NHE strategy may be a favourable option for elderly and young female patients.

## Data Availability

The datasets generated and/or analysed during the current study are not publicly because we will enlarge the sample size and extend the follow-up time to further explore the relationship between NHE and clinical outcome in THA with DAA, but these are available from the corresponding author on reasonable request.

## Abbreviations

THA: Total hip arthroplasty
DAA: Direct anterior approach
THE: Traditional hip extension
OA: Osteoarthritis
Hb: Haemoglobin
BMI: Body mass index
ASA: American Society of Anesthesiologists
HHS: Harris Hip Score
NHE: No hip extension
OHS: Oxford Hip Score
VAS: Visual analogue scale
ICC: Interclass correlation coefficient
VTE: Venous thromboembolism
